# Supplementary figures and images for: Characterization and ligand binding properties of a fatty acid- and retinol- binding protein (Hp-FAR-2) from Heligmosomoides polygyrus
Source: PLoS Negl Trop Dis. 2025 Oct 13;19(10):e0013198. doi: 10.1371/journal.pntd.0013198 (PMC12543159; doi:10.1371/journal.pntd.0013198)

**A.**

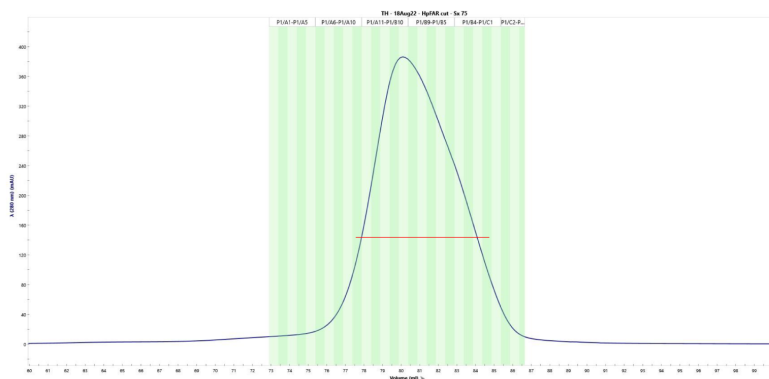

Supplement: S1 Fig — A) SEC elution profile of Hp-FAR-2 fraction. Using SEC, Hp-FAR-2 was found to contain on primary peak, highlighted in green, and the fractions collected for further analysis are indicated by the red line. B) SDS-PAGE analysis of the collected SEC fractions. The elution fractions (A8, A10, A12, B7, B5, B3, B1) are loaded alongside a molecular weight marker in (left lane) with molecular weights labeled in kDa. A prominent band around 17 kDa (shown under the red line) indicates the presence of the purified protein. (PDF) [file pntd.0013198.s001.pdf]
