# Supplementary material for: Characterization and ligand binding properties of a fatty acid- and retinol- binding protein (Hp-FAR-2) from Heligmosomoides polygyrus
Source: PLoS Negl Trop Dis. 2025 Oct 13;19(10):e0013198. doi: 10.1371/journal.pntd.0013198 (PMC12543159; doi:10.1371/journal.pntd.0013198)
Supplement: S2 Fig — A consensus casein kinase II phosphorylation site at residue 46–49 (underlined), predicted signal peptide (highlighted in grey), and predicted cleavage site (red) are shown. (PDF) [file pntd.0013198.s002.pdf]

Hp-FAR-2 MIRQIAAIAVLVCVASAGPITSIEDIPAEYRELIPKEAKDFLTGLSDADKAVLKDIAKNY 60

Hp-FAR-2 ATYKNEEEALAALKEKSPELGAKAEKLHMLVKSKIDALGEEAKVFAKEIIAGARKIQAAV 120

Hp-FAR-2 VAGNKPNLAELKEKAQKAIDKYKALSDAAKEDLQKQFPILTSVFKNEKFQKMAESLLAKN 180

Supplementary Figure 2. Sequence analysis of Hp-FAR-2. A consensus casein kinase II phosphorylation site at residue 46-49 (underlined), predicted signal peptide (highlighted in grey), and predicted cleavage site (red) are shown.
