# Supplementary material for: Characterization and ligand binding properties of a fatty acid- and retinol- binding protein (Hp-FAR-2) from Heligmosomoides polygyrus
Source: PLoS Negl Trop Dis. 2025 Oct 13;19(10):e0013198. doi: 10.1371/journal.pntd.0013198 (PMC12543159; doi:10.1371/journal.pntd.0013198)
Supplement: S3 Fig — Flies were injected with PBS, or 200 ng Hp-FAR-2 and survival was recorded for 20 days post-injection. Hp-FAR-2 injected flies show no significant difference in survival from PBS-injected flies. Survival is graphed as a Kaplan-Meir with log-rank test p value significance indicated by an asterisk. (PDF) [file pntd.0013198.s003.pdf]

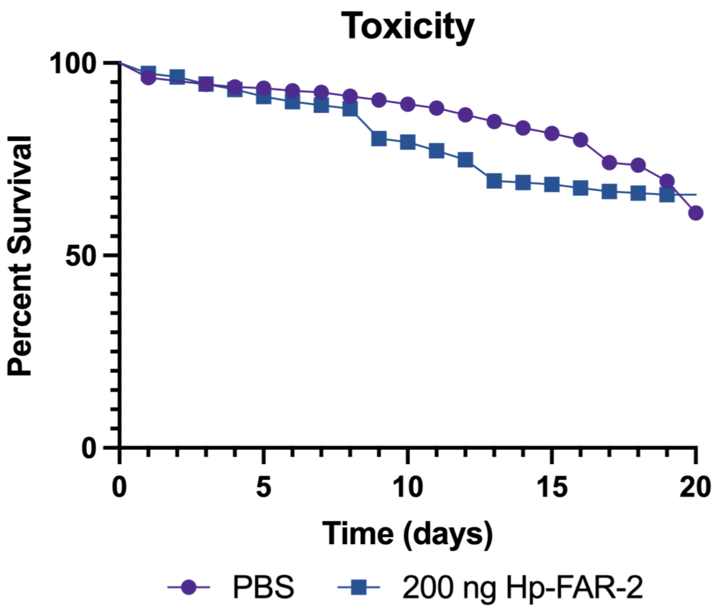

Supplementary Figure 3. Hp-FAR-2 is not toxic to *Drosophila melanogaster.* Flies were injected with PBS, or 200 ng Hp-FAR-2 and survival was recorded for 20 days post injection. Hp-FAR-2 injected flies show no significant difference in survival from PBS injected flies. Survival is graphed as a Kaplan-Meir with log-rank test p value significance indicated by an asterisk.
