# Supplementary material for: Characterization and ligand binding properties of a fatty acid- and retinol- binding protein (Hp-FAR-2) from Heligmosomoides polygyrus
Source: PLoS Negl Trop Dis. 2025 Oct 13;19(10):e0013198. doi: 10.1371/journal.pntd.0013198 (PMC12543159; doi:10.1371/journal.pntd.0013198)
Supplement: S4 Fig — Cells were incubated with Hp-FAR-2 (1 μg/ml) or PBS for 24 hours prior to exposure to pHrodo-labeled E. coli. Cells were fixed and counterstained with DAPI and subsequently imaged for analysis. A) Representative images show RAW 264.7 cells stained with DAPI (blue) and pHrodo-labeled E. coli (red), with merged channels displayed. Scale bar = 50 μm. B) Phagocytic activity was quantified as mean fluorescence intensity using ImageJ, with ~1250 cells analyzed per replicate and 4 biological replicates per treatment. Data are presented as mean + SEM. Statistical analysis was performed using an unpaired t-test in GraphPad Prism. (PDF) [file pntd.0013198.s004.pdf]

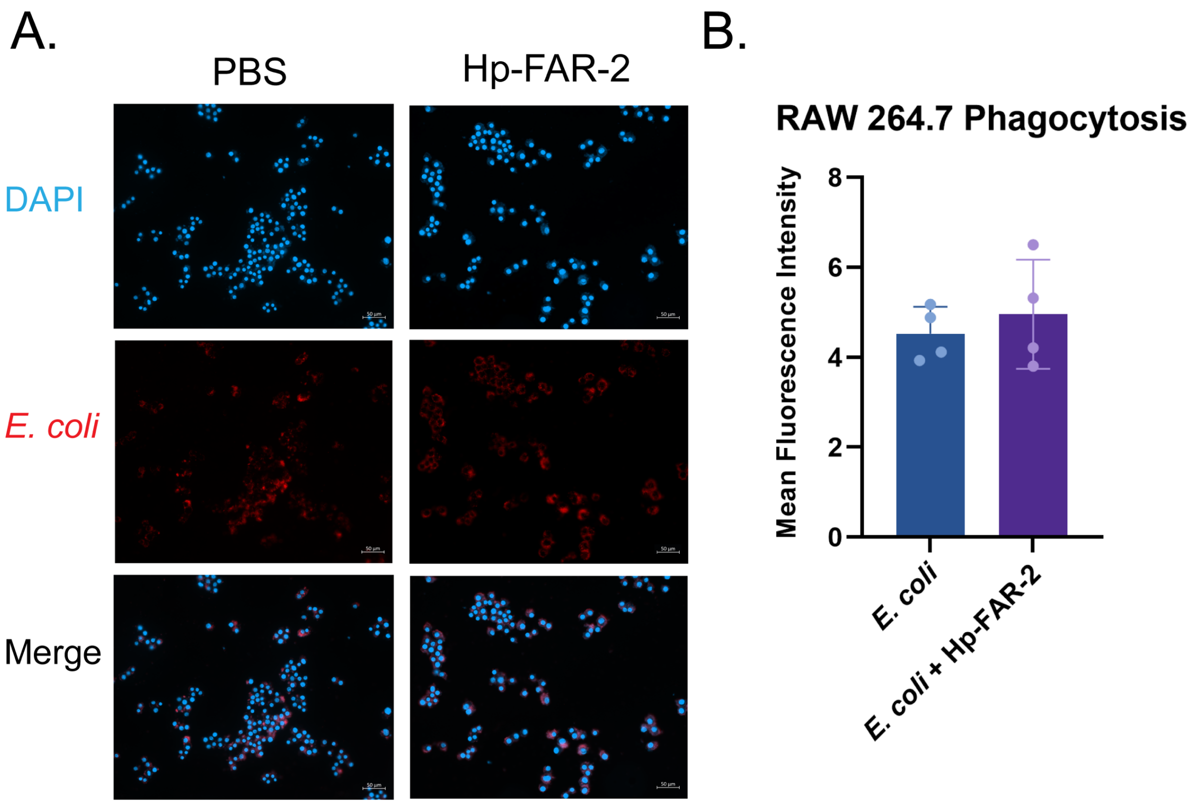

Supplementary Figure 4. Hp-FAR-2 does not affect phagocytic activity of RAW 264.7 macrophages. Cells were incubated with Hp-FAR-2 (1 µg/ml) or PBS for 24 hours prior to exposure to pHrodo-labeled *E. coli*. Cells were fixed and counterstained with DAPI and subsequently imaged for analysis. **A)** Representative images show RAW 264.7 cells stained with DAPI (blue) and pHrodo-labeled *E. coli* (red), with merged channels displayed. Scale bar = 50 µm. **B)** Phagocytic activity was quantified as mean fluorescence intensity using ImageJ, with ~1250 cells analyzed per replicate and 4 biological replicates per treatment. Data are presented as mean + SEM. Statistical analysis was performed using an unpaired t-test in GraphPad Prism.
