# Supplementary material for: Characterization and ligand binding properties of a fatty acid- and retinol- binding protein (Hp-FAR-2) from Heligmosomoides polygyrus
Source: PLoS Negl Trop Dis. 2025 Oct 13;19(10):e0013198. doi: 10.1371/journal.pntd.0013198 (PMC12543159; doi:10.1371/journal.pntd.0013198)
Supplement: S1 Table — FAR proteins were extracted from the H. polygrus genome assembly project (NCBI accession: PRJEB15396) and (NCBI accession: PRJEB1203) with WormBase ParaSite Ensembl BioMart tool, filtering for the Gp-FAR-1 domain (Pfam: 05823). The table includes gene ID, project ID, and protein name if applicable, with project PRJEB15396 and respective FAR genes labeled in red. (PDF) [file pntd.0013198.s005.pdf]

NCBI Accession ID	WormBase Parasite Gene ID	Referenced Protein name
PRJEB1203<br>PRJEB15396	HPBE_0000032401<br>HPOL_0001821001	
PRJEB1203<br>PRJEB15396	HPBE_0000435901<br>HPOL_0000227701	
PRJEB1203<br>PRJEB15396	HPBE_0001047601<br>HPOL_0000587401	
PRJEB1203<br>PRJEB15396	HPBE_0001259001<br>HPOL_0001509901	
PRJEB1203<br>PRJEB15396	HPBE_0001259101<br>HPOL_0001509801	Hp-FAR-1
PRJEB1203<br>PRJEB15396	HPBE_0002357601<br>HPOL_0001822701	Hp-FAR-2

Supplementary Table 1. List of fatty acid and retinol binding proteins (FARs) in the *H. polygrus* genome. FAR proteins were extracted from the *H. polygrus* genome assembly project (NCBI accession: PRJEB15396) and (NCBI accession: PRJEB1203) with WormBase ParaSite Ensembl BioMart tool, filtering for the Gp-FAR-1 domain (Pfam: 05823). The table includes gene ID, project ID, and protein name if applicable, with project PRJEB15396 and respective FAR genes labeled in red.
