# Supplementary material for: Characterization and ligand binding properties of a fatty acid- and retinol- binding protein (Hp-FAR-2) from Heligmosomoides polygyrus
Source: PLoS Negl Trop Dis. 2025 Oct 13;19(10):e0013198. doi: 10.1371/journal.pntd.0013198 (PMC12543159; doi:10.1371/journal.pntd.0013198)
Supplement: S2 Table — (PDF) [file pntd.0013198.s006.pdf]

Gene	Abbreviation	NCBI Gene ID	Forward Primer 5'-3'	Reverse Primer 5'-3'	Amplicon Size
Tubulin	Tub	40848	TCCACTCGTTCGGTGGAGGT	GGGCTGGGTAGATGGCGAAC	111
Drosomycin	Dros	38419	TGTTCGCCCTCTTCGCTGTC	CTGGAGCGTCCCTCCTCCTT	148
Defensin	Def	36047	CAGGCTCAGCCAGTTTCCGA	TCGCATGTGGCTCGCTTCTG	113
Metchnikowin	Metch	36708	ATGCAACTTAATCTTGGAGCGA	TGTGTTAACGACATCAGCAGTGTG	200
Diptericin	Dipt	37183	CGTCGCCTTACTTTGCTGC	CCCTGAAGATTGAGTGGGTACTG	108
Glyceraldehyde-3-phosphate dehydrogenase	GAPDH	14433	TGGCCTTCCGTGTTCCTAC	GAGTTGCTGTTGAAGTCGCA	178
Interleukin 6	IL-6	16193	CTGCAAGAGACTTCCATCCAG	AGTGGTATAGACAGGTCTGTTGG	131
Tumor necrosis factor α	TNF-α	21926	CCTGTAGCCCACGTCGTAG	GGGAGTAGACAAGGTACAACCC	148
Chitinase-like protein 3 (formerly Ym1)	Chil3	12655	CAGGTCTGGCAATTCTTCTGAA	GTCTTGCTCATGTGTGTAAGTGA	196
Arginase 1	Arg1	11846	CTCCAAGCCAAAGTCCTTAGAG	AGGAGCTGTCATTAGGGACATC	185

Supplementary Table 2. Sequences of primers used in all qPCR reactions.
