# Supplementary material for: Characterization and ligand binding properties of a fatty acid- and retinol- binding protein (Hp-FAR-2) from Heligmosomoides polygyrus
Source: PLoS Negl Trop Dis. 2025 Oct 13;19(10):e0013198. doi: 10.1371/journal.pntd.0013198 (PMC12543159; doi:10.1371/journal.pntd.0013198)
Supplement: S3 Table — (PDF) [file pntd.0013198.s007.pdf]

Ch3-UAS										
**Sequence**	**Length**	**Missed cleavages**	**Mass**	**Proteins**	**Unique (Proteins)**	**Charges**	**PEP**	**Score**	**Intensity**	**MS/MS Count**
AVLKDIAK	8	1	856.5382	HP_far	yes	2	0.051751	30.736	1709500	0
DFLTGLSDADK	11	0	1180.5612	HP_far	yes	1;2	2.69E-12	132.01	25299000	42
EAKDFLTGLSDADK	14	1	1508.7359	HP_far	yes	3	2.82E-10	79.885	5604600	15
EIIAGAR	7	0	728.41809	HP_far	yes	1	5.04E-09	116	2631600	2
IDALGEEAK	9	0	944.48148	HP_far	yes	1;2	7.84E-11	125.31	35037000	11
MAESLLAK	8	0	861.46299	HP_far	yes	1;2	1.90E-15	151.29	490480000	60
NEEEALAALK	10	0	1086.5557	HP_far	yes	1;2	1.89E-16	138.1	16523000	42
SKIDALGEEAK	11	1	1159.6085	HP_far	yes	3	0.051572	26.753	1400000	1
SPELGAK	7	0	700.37555	HP_far	yes	1	0.026562	52.231	16017000	3
**Ch2-UAS**										
**Sequence**	**Length**	**Missed cleavages**	**Mass**	**Proteins**	**Unique (Proteins)**	**Charges**	**PEP**	**Score**	**Intensity**	**MS/MS Count**
AVLKDIAK	8	1	856.5382	Hp_far_2	yes	2	0.020058	71.34	1034600	1
MAESLLAK	8	0	862.46746	Hp_far_2	yes	1;2	1.03E-21	151.29	764620000	33
NEEEALAALK	10	0	1087.5638 5	Hp_far_2	yes	2	0.0092947	92.439	1306400	2

Supplementary Table 3. Mass spectrometry data from transgenic *Drosophila* expressing Hp-FAR-2.
