# Supplementary material for: Characterization and ligand binding properties of a fatty acid- and retinol- binding protein (Hp-FAR-2) from Heligmosomoides polygyrus
Source: PLoS Negl Trop Dis. 2025 Oct 13;19(10):e0013198. doi: 10.1371/journal.pntd.0013198 (PMC12543159; doi:10.1371/journal.pntd.0013198)
Supplement: S1 Data — Macro used in ImageJ for image processing for in vivo Drosophila pHrodo assay. (PDF) [file pntd.0013198.s008.pdf]

```
Img_name=getTitle();
run("Set Measurements...", "area mean integrated limit display redirect=None decimal=3");
run("Split Channels");
close();
close();
selectWindow(img_name+" (red)");
run("Median...", "radius=2");
run("Subtract Background...", "rolling=50");
//setTool("freehand");
```

- *Select only abdomen of fly using freehand tool*

```
run("Clear Outside");
run("Measure");
run("Select None");
setAutoThreshold("Shanbhag");
//run("Threshold...");
setThreshold(30, 255);
run("Convert to Mask");
run("Analyze Particles...", "exclude summarize");
close("ROI Manager")
close("Threshold")
```

Supplementary Data 1. Macro used in ImageJ for image processing for in vivo *Drosophila* pHrodo assay.
