# Supplementary material for: Characterization and ligand binding properties of a fatty acid- and retinol- binding protein (Hp-FAR-2) from Heligmosomoides polygyrus
Source: PLoS Negl Trop Dis. 2025 Oct 13;19(10):e0013198. doi: 10.1371/journal.pntd.0013198 (PMC12543159; doi:10.1371/journal.pntd.0013198)
Supplement: Attachment — Submitted filename: PLOS_NTP_Response to Reviewers .pdf [file pntd.0013198.s010.pdf]

Response to Reviewers
PLOS Neglected Tropical Diseases

Methods

Reviewer #1: (No Response)

Reviewer #2: The objectives of the study are articulated along with a clear hypotheses of addressing the protein Hp-FAR-2 and it's functional divergence from other FAR proteins in drosophila model. I could not find information regarding the number of data points studied in the manuscript itself. I would suggest including a data section with information about the sample size.

Response to Reviewer #2 (Methods): We thank the reviewer for bringing up this point. We have included this information in the methods section, primarily towards the end of the paragraph. For further clarity we have included the total sample size. For example, we now state "For survival studies, one biological replicate is 60 flies and experiments were carried out with three biological replicates per treatment, totaling 180 flies." All methods now include total sample size for clarity.

Reviewer #3: A well focused study on a single fatty acid binding protein (Hp-FAR-2) from a widely used model helminth system, H. polygyrus, with a sophisticated Drosophila transgenesis system and some data from mouse macrophages.
Statistically all sound except Figure 5 A and C there is clearly a protective effect of Hp-FAR-2 on fly survival that perhaps an overly stringent statistical test is classifying as not significant.

Response to Reviewer #3 (Methods): The statistical test used for Figure 5A and 5C was a log-rank analysis (Mantel-Cox) which is a standard robust test used in Kaplan-Meier survival plot. This test has been used in a previous study analyzing if the treatment of a FAR protein affected survival. We thank the reviewer for bringing up this point and we have reanalyzed the survival data to ensure no missed statistical significance. We found no significant difference in survival in the FAR expressing flies (TubP>Ch2-UAS and TubP>Ch3-UAS) and their respective UAS insertion controls (Ch2-UAS/+ and Ch3-UAS/+). Similarly, the tubulin driver control (TubP>+) was also found to be insignificant compared to TubP>Ch3-UAS for females and males. However, there was a significant difference in survival between the driver control (TubP>+) and the TubP>Ch2-UAS for females and males and this has been added to the results and discussed in the paper.

Reviewer #4: The methods are appropriate to the subject of the study. Very little effect was found of HpFAR2 expression in Drosophila, but I have concerns about the experiments using recombinant protein on RAW264.7 cells. The protein was expressed in E coli, and there is no mention of removing the signal peptide, which would not be removed by expression in a prokaryotic system. It would have been better to express the protein without the signal peptide to give the functional secreted protein. More importantly, the protein was expressed in E coli, and it was not stated if endotoxin levels were measured. E coli expressed proteins are very often purified with contaminating endotoxin, and as RAW264.7 cells are highly sensitive to endotoxin stimulation, it could well be that the effects of HpFAR2 addition are due to endotoxin, rather than the protein itself. These experiments should be repeated with protein which has been shown to be endotoxin free, and a suitable control protein expressed in the same system should also be compared.

Response to Reviewer #4 (Methods): We thank the reviewer for bringing up these points. The recombinant Hp-FAR-2 expressed in *E. coli* does not have the signal peptide. We stated this in the methods as “the mature Hp-FAR-2 protein (amino acids 18 to 180 of the full-length gene)” indicating the signal peptide was not included. We have modified this sentence in the methods to further clarify the signal peptide was not included in the expressed protein. We have not included a control protein in this study but have noted this as a limitation in the revised manuscript and that future studies should include a control protein. Furthermore, we quantified the amount of endotoxin from the Hp-FAR-2 aliquot used for the RAW 264.7 cell assays using the Pierce Chromogenic Endotoxin Quant Kit (Thermo Scientific, Catalog # A39552) and found the amount to be <0.01 EU/mL which is now mentioned in the methods of the revised manuscript.

Results

Reviewer #1: (No Response)

Reviewer #2: The analysis presented matches the analysis plan, although I would urge the authors to improve clarity in language for the analysis plan. Figure 7(a) for E. coli isn't sufficiently clear for publication.

Response to Reviewer #2 (Results): We thank the reviewer for bringing up these points. We have modified the manuscript and have combined the results and discussion. This will not only condense the length of the paper but help improve clarity of the language in the results as suggested. The high-quality image of Figure 7 can be downloaded and viewed from the upper right-hand corner of the proof which is clearer than what is displayed in the master copy. We believe this high-quality version has sufficiently clear panels of the *E. coli* for publication.

Reviewer #3: The results are presented in reasonable fashion but several figures could be more clearly annotated and there are a number of questions and deficiencies.

(1) In Figure 1, the coloring is confusing, indicating greater identify than actually is the case, some errors (eg KE at 36-37 not colored), and starkly different residues given same color (eg Phe to Ala or Ile at 56/57). All substitutions have a functional significance. Better to show identities only.

Response to Reviewer #3 (Results): We thank the reviewer for the suggestions and have modified the figure as suggested. Figure 1B now displays identical residues which are shaded in orange.

(2) Line 446 and elsewhere, the mass of 19.5968 kDa is (a) false accuracy and (b) refers to the full length protein including the signal peptide that does not exist outside the protein synthesis machinery.

Response to Reviewer #3 (Results): We thank the reviewer for this point and have modified reporting the mass to only include the mature protein (without the signal peptide) and to be within two significant figures.

(3) Line 453 and elsewhere 91.11% identity is another example of false accuracy. The amino acid identity is over 180 residues so each difference counts for 0.55%, ie one less identity and the difference would be 90.55%, so no more than 3 significant figures are appropriate.

Response to Reviewer #3 (Results): We have changed the percent identities to be within two significant figures.

(4) Figure 2 should include a negative control protein to show the background level of nonspecific DAUDA binding.

Response to Reviewer #3 (Results): We appreciate the reviewer's suggestion to include a negative control protein in the DAUDA binding assay to account for nonspecific DAUDA binding. The fluorescence properties of DAUDA strongly depend on the polarity and accessibility of protein binding sites, and this assay has been widely validated in studies of FARs and other parasitic lipid-binding proteins (Kennedy et al. 1997, DOI: 10.1074/jbc.272.47.29442; Garofalo et al. 2002, DOI: 10.1016/s0166-6851(02)00097-x; Parks et al. 2021, DOI: 10.1371/journal.ppat.1010027; Jordanova et al. 2009, DOI: 10.1074/jbc.M109.022731). DAUDA exhibits minimal fluorescence enhancement in the presence of non-lipid-binding proteins due to the absence of a suitable hydrophobic binding pocket. Therefore, the observed fluorescence shift and intensity in our assay are consistent with specific DAUDA binding to Hp-FAR-2 and are unlikely to result from nonspecific protein interactions. We have noted in the revised manuscript that future studies should include a control protein. We included multiple controls in Figures 2A and 2C, including DAUDA and PBS alone, and have now added protein alone as an additional control.

(5) Figure 3 shows 1:1 and 1:10 ratios, but this is quite coarse grained, is there any reason not to do a full titration to better quantify the "binding preference"? The Figure should spell out names of the fatty acids tested.

Response to Reviewer #3 (Results): We appreciate the feedback regarding Figure 3. Previous studies on FARs have assessed fatty acid binding preference by measuring the displacement of DAUDA in the presence of another fatty acid, traditionally using a 1:10 fatty acid to DAUDA ratio. (Basavaraju et al. 2003, DOI: 10.1016/S0166-6851(02)00253-0; Bathe et al. 2009, PMID: 22736819; Zhan et al. 2018, DOI: 10.1371/journal.pntd.0006772; Cheng et al. 2013, DOI: 10.1371/journal.pone.0066011). We have chosen to follow this same protocol while also testing a more competitive 1:1 ratio to improve resolution of binding preference and have expanded the assay to include seven different fatty acids. We believe for the purpose of identifying fatty acid preference these assays are sufficient and have gone beyond the standard assay protocol commonly used in previous FAR studies. We have changed Figure 3 to spell out the fatty acids as suggested.

(6) Also clarify annotations in Figure 5, "TunP>Ch2-UAS" takes quite some deciphering, and colors on the line graph are too similar to discern.

Response to Reviewer #3 (Results): We appreciate the feedback regarding Figure 5. The annotations used in Figure 5 are the standard *Drosophila* annotations to represent the product of a GAL4/UAS cross. For example, TubP>Ch2-UAS denotes a cross between the Hp-FAR-2 UAS insertion on the second chromosome with the tubulin promoter (TubP), driving ubiquitous expression of Hp-FAR-2 throughout the fly. We understand these annotations are confusing and have made sure to clearly explain them in the results/discussion and figure legend for the reader. Furthermore, we have changed the color scheme for Figure 5 to make each line easily distinguishable from each other.

(7) In general not clear how doses were selected, for example only a single injection of 200 ng of Hp-FAR-2 was tested for effects over 20 days; macrophages were cultured with 3 ug/ml.

Response to Reviewer #3 (Results): A single injection of 200 ng of recombinant protein is physiologically relevant for the *Drosophila* infection model used in this study, where biological responses can be observed without causing overwhelming toxicity to the fly. A previous study using *Drosophila* injected similar amounts of FAR protein, with doses ranging from 20 ng to 250 ng (Parks et al. 2021, DOI:

10.1371/journal.ppat.1010027). We injected a one-time dose of 200 ng Hp-FAR-2 and observed no effect on fly survival. Furthermore, the dose of 3ug/mL for macrophages is within the physiologically relevant range of parasite excretory/secretory products in an infected mouse and this was mentioned in the discussion section. Our goal in choosing 200 ng was to use a dose at the high end of what was physiologically relevant.

(8) It is not clear whether key data on macrophages (Figure 8) represents repeated experiments, ie were the "biological replicates" all in the same experiment, or was this result obtained from ≥2 independent experiments, one of which is represented in the Figure? The Legend does not specify timing, there is no control protein added, and the measures are restricted to PCR for mRNA (and hence does not warrant the term "marker" which implies measurement of cell surface moieties by flow cytometry).

Response to Reviewer #3 (Results): We appreciate the reviewers thoughtful feedback regarding Figure 8 which presents data from one independent experiment, in which three biological replicates of cells were tested per treatment group. We have modified this information in the methods and legend to avoid further confusion. We have also included the timing (48-hour incubation period) in the legend. For this experiment, the negative control was vehicle (PBS), and we acknowledge the reviewer's point regarding the absence of a negative control protein. We agree that a control protein would help distinguish specific from non-specific effects. Due to technical constraints, a control Hp-FAR-2 protein was not included in this study; however, this remains an important direction for future work and has been acknowledged in the revised manuscript. We have modified the titles of Figure 8 to say expression of M1/M2 markers for clarity .

Reviewer #4: Data presented in an appropriate manner

Response to Reviewer #4 (Results): We thank the reviewer for their comment.

Conclusions

Reviewer #1: (No Response)

Reviewer #2: Limitations of analysis such as the doses of hp-FAR-2 are addressed in the manuscript. Authors also address the role Hp-FAR-2 may play in lipid absorption and modulate expression of other lipids.

Response to Reviewer #2 (Conclusions): We thank the reviewer for their comments.

Reviewer #3: In general, the conclusions are matched by the data, but the key finding (quoted in the title) that the protein "suppresses host macrophage polarization" is not well supported, with a single experiment (Figure 8) at a single dose using only RT-PCR and no control protein. This should be repeated and extended over a dose range and coupled with flow cytometry.

Response to Reviewer #3 (Conclusions): We agree that testing across a broader dose range, using a control protein, and incorporating flow cytometry would further strengthen the macrophage polarization experiment. However, these experiments are beyond the scope of the current study, and we have acknowledged these limitations in the discussion. The title has been changed accordingly to reflect a better representation of the paper as suggested.

Reviewer #4: Throughout the manuscript, including the abstract, it is stated that Omega3/6 fatty acids are immunoactive, however little evidence is presented for this. The reference for Oleic acid (the target with the best evidence for binding) being immunoactive is a single review article, and is not conclusive.

In the discussion, it is suggested that the lipid binding ability of HpFAR2 could switch host cells to production of immunoactive lipids such as the prostaglandins, leukotrienes etc. However these are all arachidonic acid metabolites, which HpFAR2 does not seem to preferentially bind to. This should be discussed.

Response to Reviewer #4 (Conclusions): We appreciate the feedback regarding citing evidence of omega-3 and omega-6 being immunoreactive lipids in the paper. Due to the already lengthy discussion and introduction we chose not elaborate on this topic. However, as suggested, we have now included this information in the revised manuscript and cited studies demonstrating evidence of oleic acid immunoreactivity in the discussion

It is important to note that although Hp-FAR-2 does not exhibit a preference for arachidonic acid (AA) in the 1:1 competition assay, this does not imply that Hp-FAR-2 fails to bind AA in the host. In fact, Figure 3A demonstrates that Hp-FAR-2 binds to both LA and AA in a 1:10 competition ratio with DAUDA, which provides evidence that Hp-FAR-2 may sequester LA/AA to influence eicosanoid production in the host.

Editorial and Data Presentation Modifications

Reviewer #1: (No Response)

Reviewer #2: Minor revisions needed. The manuscript tries to cover too much ground, making it difficult to identify the key findings and relate the data to findings. Other revisions mentioned above.

Response to Reviewer #2 (Editorial and Data Presentation Modifications): We appreciate all the revisions mentioned in the previous sections. We have combined the results and discussion which condenses the manuscript and improves the overall coherence and flow, making it easier for readers to identify the key findings and relate them to the data.

Reviewer #3: The manuscript is very, very wordy, with the Discussion repeating much of the results; a substantial reduction in length would be appropriate, as would moving some of the negative data (eg figure 7) to supplementary information.

Response to Reviewer #3 (Editorial and Data Presentation Modifications): We appreciate the feedback and have combined the results and discussion into a single section to avoid repetition and condense the length of the paper. Furthermore, we have moved Figure 7 to the supplementary materials, as suggested.

Reviewer #4: Line 61-65: this sentence is unclear (double negative) and should be rewritten.

Response to Reviewer #4 (Editorial and Data Presentation Modifications): We have rewritten the sentence as suggested.

Summary and General Comments

Reviewer #1: General comments
The paper deals with a family of proteins from nematodes, the FAR proteins, that are being increasingly recognised for their importance in parasitic nematodes controlling the immune defence reactions of their hosts. Such effects mediated by FAR proteins have been found in nematode infections of plants and animals. Understanding how these proteins have their effects is potentially important for developing means by which the health of humans, domestic animals, and crop plants could be improved. The paper

aims to examine the effect of a FAR protein (Hp-FAR-2) from a parasitic nematode of mice, Heligmosomoides polygyrus, a parasite with well-described immunomodulatory properties. But most of the experiments reported examine the influence of the protein in Drosophila melanogaster insects rather than in a mammalian system. This approach is imaginative but assumes that the FAR protein concerned will operate similarly across phyla of hosts, even though it has similar lipid-binding properties to most other FAR proteins so far described. The authors have previously shown that a FAR from an insect parasite does have effects on Drosophila, which could be because that protein engages in interactions beyond the binding of lipids. In the present paper, however, the Hp-FAR-2 protein is shown to have no effects on immune system parameters in flies or on their survivability in bacterial infections. The authors then jump to examining the effect of Hp-FAR-2 on macrophages from mice in the search for some effects. The paper therefore reads like two separate papers – one that shows no effects in flies, then one that shows some effects on a completely different host system – the latter being on the face of it a more straightforward (though with much room for development) examination of the effects of a protein from a parasite of mice on an aspect of the murine immune system. This juxtaposition does seem confusing.

Specific points.
1. The title does not properly represent the paper's content. As said above, the bulk of the paper deals with Hp-FAR-2 in Drosophila and that the mouse macrophage aspect is tacked on the end, yet the title mentions only the latter.

Response to Reviewer #1 (Summary and General Comments): The title has been changed accordingly to reflect a better representation of the paper as suggested.

2. Line 70 – citation [34] refers to a FAR protein (entirely appropriate to the paper) but not to a different class of proteins from nematodes as intended. I did a search and see that the same group as mentioned in that citation published on the polyprotein type - doi.org/10.1371/journal.pntd.0001040 , which is perhaps what is intended?

Response to Reviewer #1 (Summary and General Comments): We appreciate the reviewer for bringing up this point and providing a more informative citation regarding nematode polyproteins allergens. We have modified that citation accordingly.

3. Line 274 – Is this statement true for all FAR proteins?

Response to Reviewer #1 (Summary and General Comments): FAR proteins have a ligand-binding pocket for fatty acids which can be assessed using the fluorescent fatty acid DAUDA. The fluorescence properties of DAUDA strongly depend on the polarity and accessibility of protein binding sites. DAUDA exhibits minimal fluorescence in the presence of non-lipid-binding proteins due to the absence of a suitable hydrophobic binding pocket. This assay has been widely validated in studies of FARs and other parasitic lipid-binding proteins (Kennedy et al. 1997, DOI: 10.1074/jbc.272.47.29442; Garofalo et al. 2002, DOI: 10.1016/s0166-6851(02)00097-x; Parks et al. 2021, DOI: 10.1371/journal.ppat.1010027; Jordanova et al. 2009, DOI: 10.1074/jbc.M109.022731).

4. Line 464 – What is "the FAR protein domain"?

Response to Reviewer #1 (Summary and General Comments): The FAR protein domain refers to the conserved protein domain found in FARs specialized for binding fatty acids and retinol. The term FAR protein domain can be classified as a pfam domain pfam05823:Gp-FAR-1 (Jordanova, 2009, DOI: 10.1074/jbc.M109.022731).

5. Figure 1B – If included, it would be useful to indicate the cleavable secretory signal peptides in the alignment.

Response to Reviewer #1 (Summary and General Comments): We thank the reviewer for the suggestion. The cleavable secretory signal peptide for Hp-FAR-2 has been highlighted in the supplementary information (see Supplementary Figure 2).

6.Figures 2A and 2C – The spectra look remarkably noise-free – have they been smoothed? Also, in Materials and Methods it is stated that the spectra are corrected for background fluorescence of PBS, presumably by subtraction, but the signal for PBS is included and is higher in places than the spectra with DAUDA +/- Hp-FAR-2, presumably because it is the uncorrected version? Would it not be more straightforward merely to show all the spectra uncorrected? Incidentally, the signal for PBS alone is quite high, particularly at the shorter wavelengths – is there a reason for that?

Response to Reviewer #1 (Summary and General Comments): The data points for Figure 2 were normalized to the peak fluorescence intensity of DAUDA to FAR (yielding a value of 1) and corrected for background fluorescence of PBS alone, hence why they look smoothed. The signal for PBS was only normalized to peak fluorescence and was chosen to be included in the figure as it is a control. We agree that the previous presentation may have been confusing, and that displaying the raw data is more straightforward. We now present the raw data for all groups uncorrected, with values normalized to 1. The spectra for PBS is a broad peak around ~400 nm due to Raman scattering by water. This can be seen in other FAR papers performing the same assay (Kennedy et al. 1997, DOI: 10.1074/jbc.272.47.29442; Prior et al. 2001. DOI: 10.1042/0264-6021:3560387; Bathe et al. 2009, PMID: 22736819).

7. Figure 2B – the fitted curve does not fit the data points well. Is this because of the aberrant last point? If so, might its removal improve the fit? Other than improving the affinity calculation it could improve the statement about relative affinity with retinol.

Response to Reviewer #1 (Summary and General Comments): We thank the reviewer for the suggestion. We have removed the last point as suggested and it has improved the fit significantly which may accurately calculate the affinity as mentioned.

8. Figure 2D – The curve fit is not ideal for retinol either, and the data points are very scattered. Retinol can be subject to decay in water – has this been compensated for? In lines 483 and 484 it is stated that "These values reveal that relative to retinol, Hp-FAR-2 has a higher affinity for fatty acids." Care might be needed here because it could be risky to generalise to fatty acids from a calculation based on a fatty acid with quite a large fluorophore attached. Also, given the above comments on the curve fitting, and the intrinsic errors involved, the calculated affinities for DAUDA and retinol are well within an order of magnitude, so may not be meaningfully different.

Response to Reviewer #1 (Summary and General Comments): We thank the reviewer for the insightful comments. In the methods we state that retinol was freshly diluted to a working concentration and used within 24 hours of preparation of stock, and this was done to reduce decay. We do use relatively high concentrations of retinol (up to 30 uM) to create the fitted curve, and it is likely that retinol at these higher concentrations (>20 uM) has decayed and adversely affected the binding curve. We have excluded these data points of the retinol binding curve (22 μM to 30 μM), which we believe have significantly improved the curve fit. Furthermore, we agree with the statement regarding the difference in affinities as they are in the same magnitude and it has been removed from the discussion.

9. The lipid preferences of the protein show, in Figure 3 reveal a surprise in that Hp-FAR-2 shows little or no affinity for linolenic acid. Is there any precedent for this seeming high degree of specificity in this protein family, and do the authors have any suggestion for how it comes about?

Response to Reviewer #1 (Summary and General Comments): We thank the reviewer for this insightful question. We believe the relatively low affinity of Hp-FAR-2 for alpha-linolenic acid (ALA) could be related to the fact that ALA is less abundant in mammalian plasma relative to other polyunsaturated fatty acids. One study measuring total plasma lipids in humans found that ALA concentrations were significantly lower than linoleic acid (approximately 180 µmol/L vs. 5.0 mmol/L, respectively) (Abdelmagid et al. 2015, DOI: 10.1371/journal.pone.0116195). It is possible that there is less evolutionary pressure for Hp-FAR-2 to bind to ALA strongly and it may have adapted to bind to more abundant fatty acids such as linoleic acid in the host.

10, Lines 481-482 – "The Kd for Hp-FAR-2:retinol was determined by increasing the concentration of retinol (0-30 µM) in the presence of 1 µM DAUDA" – is this correct?

Response to Reviewer #1 (Summary and General Comments. No, this is not correct. The sentence is meant to read, "*The Kd for Hp-FAR-2:retinol was determined by increasing the concentration of retinol (0-30 µM) in the presence of 1 µM Hp-FAR-2".* We thank the reviewer for bringing this to our attention and it has now been corrected.

11. Figure 5 – Understanding the list of experimental groups to the right in the figure requires considerable reference to the figure's legend and the main text. Can it be simplified to make clear what each experimental group represents?

Response to Reviewer #1 (Summary and General Comments): We appreciate the feedback regarding Figure 5. The annotations used in Figure 5 are the standard *Drosophila* annotations to represent the product of a GAL4/UAS cross. We understand these annotations can be difficult for the reader to reference and have made sure to explain them as clearly as possible in the results/discussion and figure legend for the reader.

12. Figure 7A&B – This figure shows that Hp-FAR-2 does not enhance phagocytosis. But might this be a case for adding a positive control protein to demonstrate that the assay is working?

Response to Reviewer #1 (Summary and General Comments): We believe the nature of the pHrodo assay itself is intrinsically self-validating. pHrodo fluorescence is minimal at neutral pH and increases only upon internalization of the labeled particles into phagosomes and/or lysosomes. Therefore, detection of increased fluorescence directly indicates successful particle uptake and acidification, confirming that the assay is working as intended.

Minor points.
1. When doing BLAST searches and the like to find similar proteins it is preferable to use the term "similarity" (which is what one searches for and identifies) rather than "homology" (which is an evolutionary inference).
2. "Data" is plural, so where it occurs it should read "data were".
3. "Primary sequence" as used in line 465 – this should be "primary structure" which, for a protein refers to its amino acid sequence. Or just say "sequence".
4. The term "technical replicates" is used a few times, but what does it mean – merely a replicate?

Response to Reviewer #1 (Summary and General Comments): We greatly appreciate the reviewer's comments and have incorporated all suggestions from the minor points section into the revised

manuscript. In our manuscript, the term “technical replicate” refers to repeated measurements of the same sample. We specifically use this term in the methods for well based assays like the fluorescent ligand-binding experiments. Technical replicates are critical in these assays to ensure reliability and consistency by minimizing variation arising from pipetting.

Reviewer #2: The research addresses an important problem with clear biomedical relevance. The experimental approach is multi-faceted, using both Drosophila infection models and mammalian cell culture to characterize the protein. The finding of functional divergence within the FAR family is noteworthy and adds to our understanding of these proteins.

Response to Reviewer #2 (Summary and General Comments): We greatly appreciate and thank the reviewer for their thoughtful comments and suggestions.

Reviewer #3: An incremental study representing a considerable body of work with Drosophila that was not so successful, and a single experiment on mouse macrophages that needs strengthening if it is to be claimed in the Title.

Response to Reviewer #3 (Summary and General Comments): We greatly appreciate and thank the reviewer for their comments. The title has been changed accordingly to reflect a better representation of the paper as suggested.

Reviewer #4: This manuscript concerns a fatty acid and retinol binding protein from H. polygyrus. it shows the binding specificity of the protein, and tests it in Drosophila and RAW264.7 macrophage cell line experiments. It is well presented and written, however the potential caveat of endotoxin contamination of purified protein must be addressed to allow the conclusions to be drawn.

Response to Reviewer #4 (Summary and General Comments): We greatly appreciate the reviewer’s comments and agree the presence of endotoxin must be addressed. We have tested for potential endotoxin contamination and have addressed this comment in the methods section.
